# Supplementary material for: An autoinhibited state of 53BP1 revealed by small molecule antagonists and protein engineering
Source: Nat Commun. 2023 Sep 29;14:6091. doi: 10.1038/s41467-023-41821-6 (PMC10541411; doi:10.1038/s41467-023-41821-6)
Supplement: Supplementary file 1 — Supplementary Information [file 41467_2023_41821_MOESM1_ESM.pdf]

Supplementary Information

An autoinhibited state of 53BP1 revealed by small molecule antagonists and protein engineering

Gaofeng Cui[1,*], Maria Victoria Botuyan[1,*], Pascal Drané[2,*], Qi Hu[1], Benoît Bragantini[1], James R. Thompson[3], David J. Schuller[4], Alexandre Detappe[5], Michael T. Perfetti[6], Lindsey I. James[6,7], Stephen V. Frye[6,7], Dipanjan Chowdhury[2] and Georges Mer[1,8,#]

*Equal contributions

[#]Correspondence: mer.georges@mayo.edu

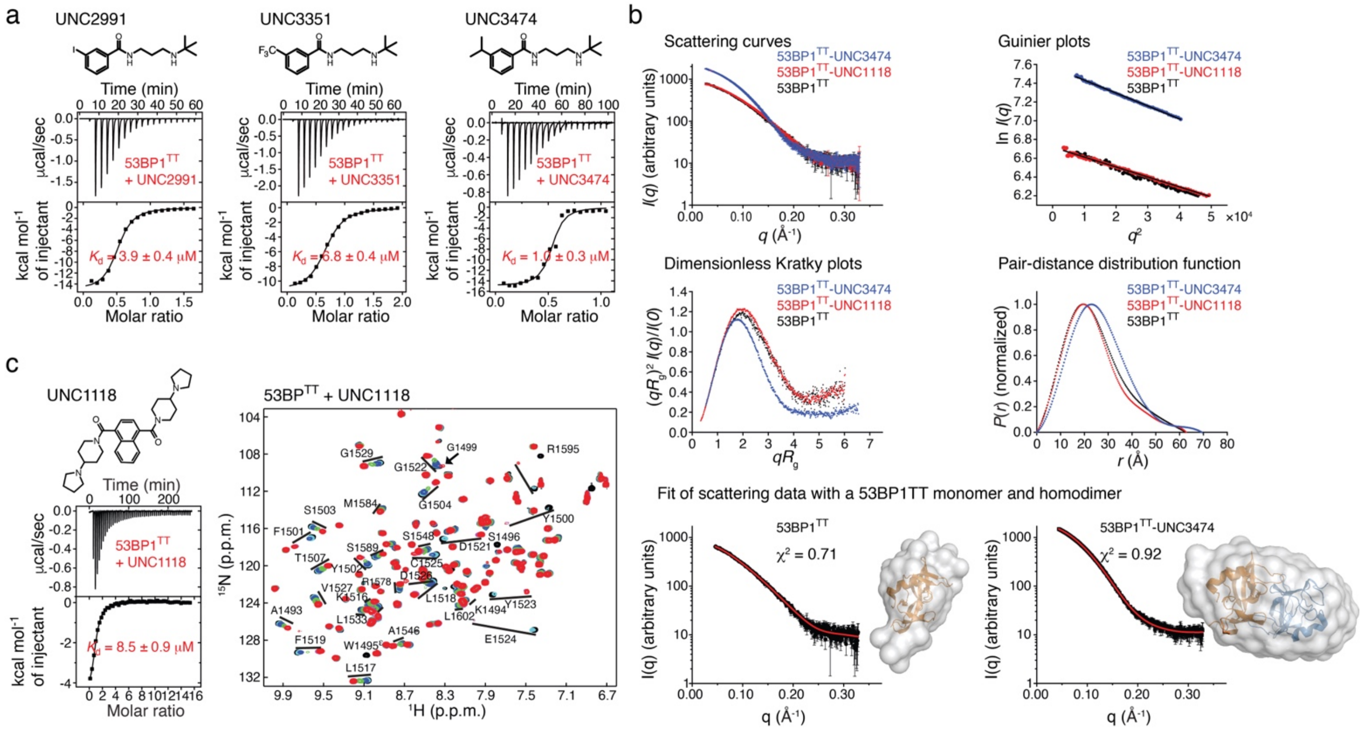

Supplementary Figure 1 | Interaction of 53BP1^{TT} with small molecules probed using isothermal titration calorimetry (ITC) and small-angle X-ray scattering (SAXS).

a, ITC of the interaction of UNC2991, UNC3351 and UNC3474 with 53BP1^{TT}. Raw titration data and integrated heat measurements are shown. Indicated apparent K_ds with associated standard deviations were determined by nonlinear least-squares analysis.

b, SAXS curves for 53BP1^{TT}, 53BP1^{TT}-UNC1118 and 53BP1^{TT}-UNC3474. 53BP1^{TT} was used at 0.67, 1.33 and 2 mg/mL with an exposure time of 0.5 s, without and with 10-fold molar excess of UNC1118 or UNC3474. The error bars in the scattering curves were generated using PRIMUS in the data analysis software ATSAS 2.4.2 and represent mean ± standard deviation calculated from signal intensity scaling and merging for the three different 53BP1^{TT} concentrations. The Guinier plots, dimensionless Kratky plots and pair distance distribution functions derived from the SAXS data are shown. Also shown are the crystal structures of 53BP1^{TT} and 53BP1^{TT}-UNC3474 overlaid to the corresponding SAXS-based *ab initio* molecular envelopes generated using GASBOR 2.3i.

c, Interaction of UNC1118 with 53BP1^{TT} characterized using ITC (left) and NMR spectroscopy (right). For the ITC, raw titration data and integrated heat measurements are shown. The indicated K_d with associated standard deviation was determined by nonlinear least-squares analysis.

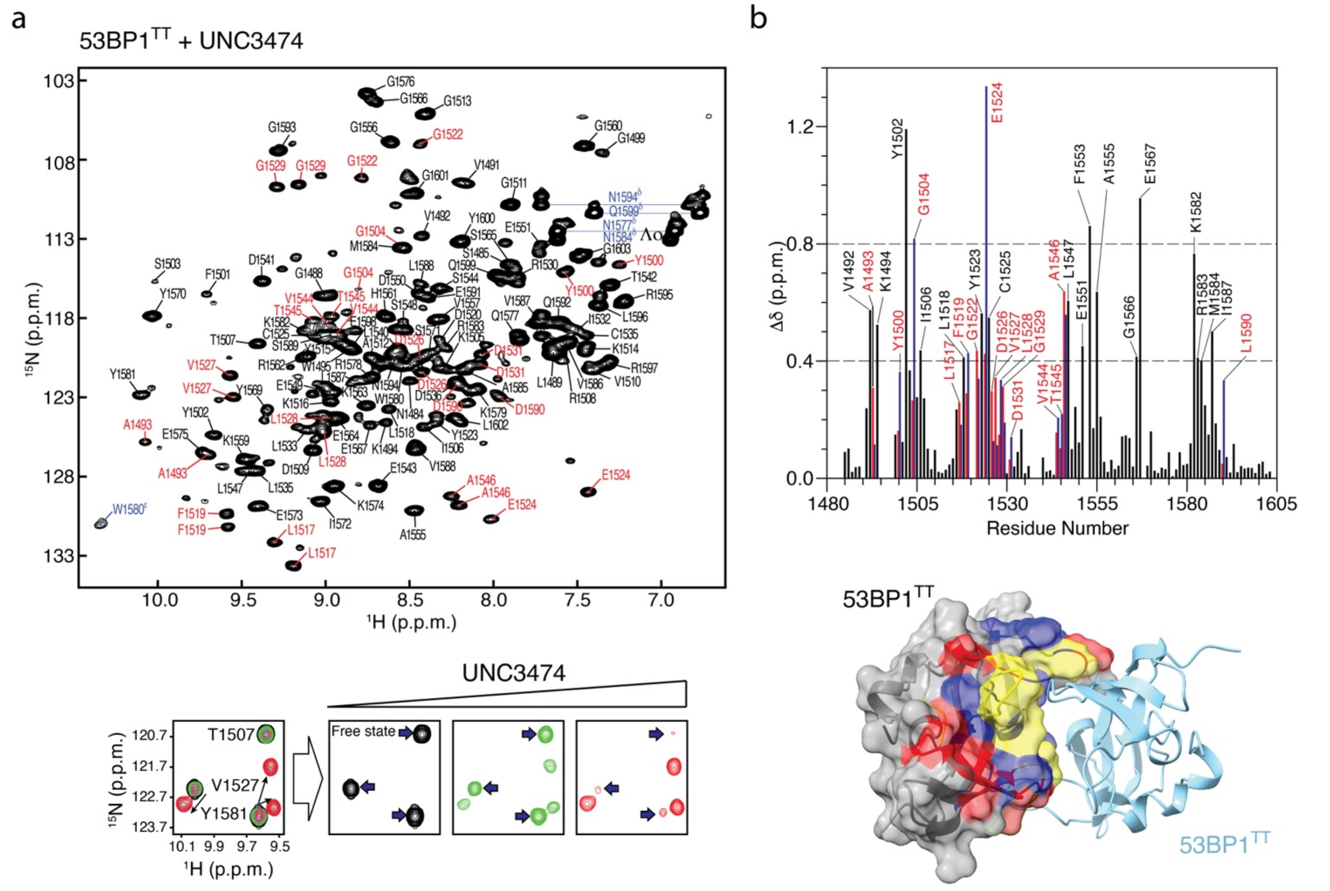

Supplementary Figure 2 | Interaction of 53BP1^{TT} with small molecules probed using NMR spectroscopy.

a, Top: Assigned ^{1}H-^{15}N HSQC spectrum of ^{15}N-labeled 53BP1^{TT} bound to non-labeled UNC3474 at a 53BP1^{TT}:UNC3474 molar ratio of 1:10. Residues for which there is doubling of resonances are labeled in red. The ^{1}H-^{15}N resonances for the asparagine and glutamine side chain amide groups and tryptophan indole ring are labeled in blue. Bottom: Changes in NMR signal positions for three residues in the ^{1}H-^{15}N HSQC spectrum of ^{15}N-labeled 53BP1^{TT} upon titration with non-labeled UNC3474 highlighting the slow exchange on the chemical shift time scale.

b, Top: Chemical shift perturbations (Δδ) in ^{15}N-labeled 53BP1^{TT} at 10-fold molar excess of non-labeled UNC3474. Black bars are for residues with Δδ > 0.4. Blue and red bars are for residues with split signals. Bottom: Mapping of chemical shift perturbations on the surface and ribbon representation of one of the two protomers in the 53BP1^{TT}-UNC3474 structure (UNC3474 omitted). Residues with Δδ > 0.4 are colored blue and those with split signals are colored red. Residues for which signals are missing due to exchange broadening are colored yellow.

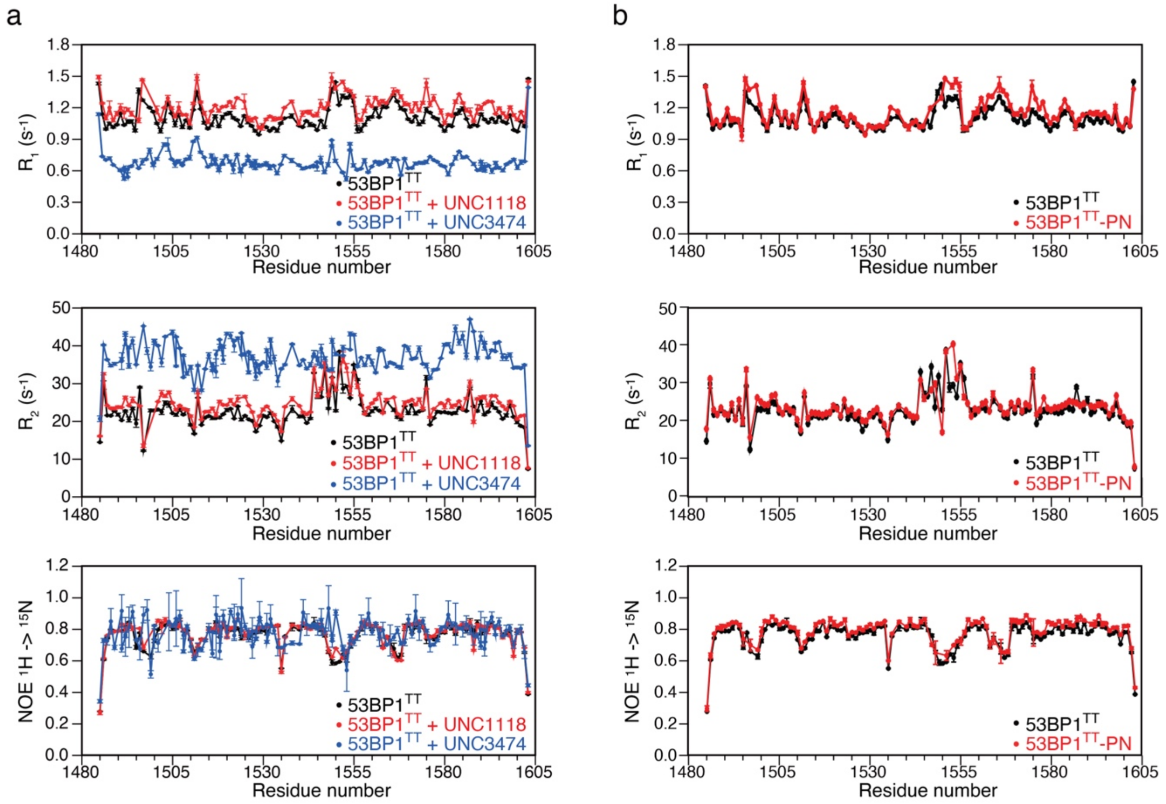

Supplementary Figure 3 | NMR relaxation measurements.

a, NMR relaxation values for 53BP1^{TT} without and with 9-fold molar excess each of UNC3474 and UNC1118. The R_1 and R_2 values were calculated using NMRViewJ with errors determined via relaxation curve fitting. For ^{15}N-{^{1}H} NOEs, shown are the average values ± standard deviation calculated from two independent sets of measurements.

b, NMR relaxation values for 53BP1^{TT} and 53BP1^{TT}-PN obtained as in **a**.

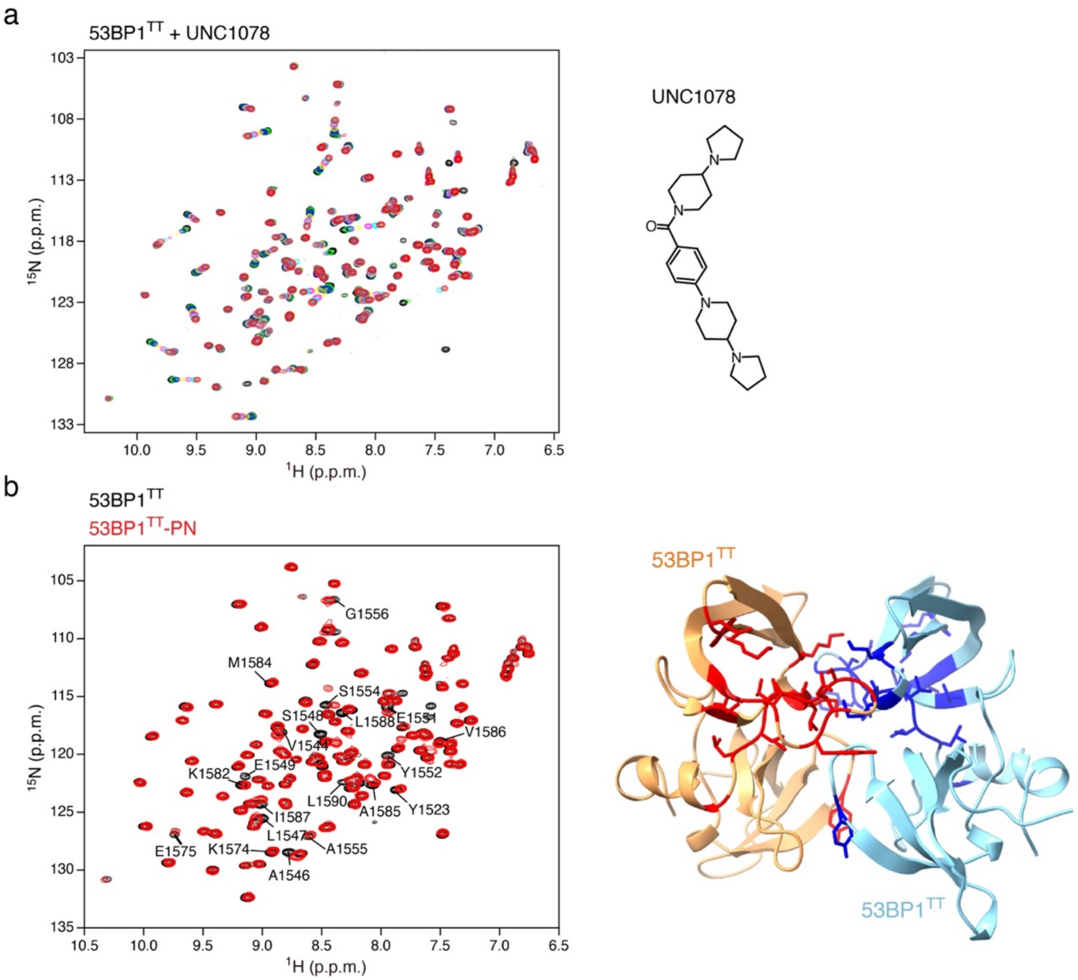

Supplementary Figure 4 | Comparative NMR spectroscopy of 53BP1^{TT} and 53BP1^{TT}-PN.

a, NMR spectroscopy-monitored interaction of 53BP1^{TT} with UNC1078. Shown in different colors are the overlaid ^{1}H-^{15}N HSQC spectra of 53BP1^{TT} recorded without (black) and with increasing amounts of UNC1078, up to 4-fold molar excess (red).

b, Overlay of the ^{1}H-^{15}N HSQC spectra of 53BP1^{TT} and 53BP1-^{TT}-PN. The two proteins were at a concentration of 0.3 mM. Residues for which the chemical shifts changed are labeled on the left. The residues are mapped (in red and blue colors) on the structure of the 53BP1^{TT} homodimer bound to UNC3474 (UNC3474 omitted) on the right.

Supplementary Table 1 | SAXS reporting data.

**Sample details**			
	53BP1^{TT}	53BP1^{TT}-UNC1118	53BP1^{TT}-UNC3474
Organism	*Homo Sapiens*	*Homo Sapiens*	*Homo Sapiens*
Source	*E. coli*	*E. coli*	*E. coli*
Description: sequence & ligands	Q12888 (53BP1 residues 1484-1603)	Q12888 (53BP1 residues 1484-1603), UNC1118	Q12888 (53BP1 residues 1484-1603), UNC3474
Extinction coefficient ε (wavelength and units)	280 nm, 24,410 M^{-1} cm^{-1}	280 nm, 24,410 M^{-1} cm^{-1}	280 nm, 24,410 M^{-1} cm^{-1}
Molecular mass (Da)	13,925.79	13,925.79	13,925.79
Concentration (range/values) measured	0.67, 1.33, 2 mg/mL	0.67, 1.33, 2 mg/mL	0.67, 1.33, 2 mg/mL
Solvent composition	25 mM sodium phosphate, pH 7.5, 15 mM NaCl	25 mM sodium phosphate, pH 7.5, 15 mM NaCl	25 mM sodium phosphate, pH 7.5, 15 mM NaCl
**SAXS data collection parameters**			
Source	SIBYLS beamline 12.3.1 at the Advanced Light Source		
Wavelength (Å)	1.127		
Beam geometry (size, sample-to-detector distance)	0.1 x 0.1 mm, 1.5 m		
q-measurement range ($Å^{-1}$)	0.013-0.33		
Method for monitoring radiation damage	Varied exposure lengths were compared against one another to assess damage		
Exposure time, number of exposures	0.5 s, 1 s, 2 s exposures for each concentration		
Sample configuration	A 1 mm sample was placed between two mica windows in a transmission geometry		
Sample temperature	10 ºC		
**Software employed for SAXS data reduction, analysis and interpretation**			
SAS data reduction	Subtraction using established programs at SIBYLS beamline 12.3.1		
Basic analyses and merging of curves.	PRIMUS from ATSAS 2.4.2		
Shape/bead modeling	GASBOR 2.3i		
**Structural parameters**			
Guinier analysis			
	53BP1^{TT}	53BP1^{TT}-UNC1118	53BP1^{TT}-UNC3474
$I(0)$ (in detector units)	847.5 ± 6.2	829.6 ± 3.3	1918.7 ± 5.4
R_g (Å)	19.1 ± 0.2	17.9 ± 0.1	20.1 ± 0.1
q-range ($Å^{-1}$)	0.027-0.33	0.018-0.33	0.027-0.33
qR_g max	1.3	1.3	1.3
$P(r)$ analysis			
	53BP1^{TT}	53BP1^{TT}-UNC1118	53BP1^{TT}-UNC3474
$I(0)$ (in detector units)	832.0 ± 3.1	833.3 ± 2.1	1919.0 ± 3.1
R_g (Å)	18.85 ± 0.069	18.34 ± 0.061	20.18 ± 0.038
d_{max} (Å)	62.5	62.7	69.4
q-range ($Å^{-1}$)	0.01-0.33	0.01-0.33	0.01-0.33
Porod volume ($Å^3$)	21822	22413	37692
**Shape modelling results**			
	53BP1^{TT}	53BP1^{TT}-UNC3474	
GASBOR 2.3i			
q-range for fitting	0.027-0.33	0.027-0.33	
Symmetry	P1	P1	
χ^2 value	0.71	0.92	
d_{max} (Å), R_g (Å)	62.55, 18.37	69.45, 19.90	
