# Supplementary material for: An autoinhibited state of 53BP1 revealed by small molecule antagonists and protein engineering
Source: Nat Commun. 2023 Sep 29;14:6091. doi: 10.1038/s41467-023-41821-6 (PMC10541411; doi:10.1038/s41467-023-41821-6)
Supplement: Supplementary file 3 — Reporting Summary [file 41467_2023_41821_MOESM3_ESM.pdf]

nature portfolio

Corresponding author(s): Georges Mer

Last updated by author(s): Sep 13, 2023

Reporting Summary

Nature Portfolio wishes to improve the reproducibility of the work that we publish. This form provides structure for consistency and transparency in reporting. For further information on Nature Portfolio policies, see our Editorial Policies and the Editorial Policy Checklist.

Statistics

For all statistical analyses, confirm that the following items are present in the figure legend, table legend, main text, or Methods section.

n/a	Confirmed	
☐	☒	The exact sample size (*n*) for each experimental group/condition, given as a discrete number and unit of measurement
☐	☒	A statement on whether measurements were taken from distinct samples or whether the same sample was measured repeatedly
☐	☒	The statistical test(s) used AND whether they are one- or two-sided<br>*Only common tests should be described solely by name; describe more complex techniques in the Methods section.*
☒	☐	A description of all covariates tested
☒	☐	A description of any assumptions or corrections, such as tests of normality and adjustment for multiple comparisons
☐	☒	A full description of the statistical parameters including central tendency (e.g. means) or other basic estimates (e.g. regression coefficient) AND variation (e.g. standard deviation) or associated estimates of uncertainty (e.g. confidence intervals)
☐	☒	For null hypothesis testing, the test statistic (e.g. *F*, *t*, *r*) with confidence intervals, effect sizes, degrees of freedom and *P* value noted<br>*Give P values as exact values whenever suitable.*
☒	☐	For Bayesian analysis, information on the choice of priors and Markov chain Monte Carlo settings
☒	☐	For hierarchical and complex designs, identification of the appropriate level for tests and full reporting of outcomes
☒	☐	Estimates of effect sizes (e.g. Cohen's *d*, Pearson's *r*), indicating how they were calculated

Our web collection on statistics for biologists contains articles on many of the points above.

Software and code

Policy information about availability of computer code

Data collection	X-ray diffraction data were collected at the 19-BM and 19-ID beamlines of the Advanced Photon Source at Argonne National Laboratory, IL and at beamline A1 of the Cornell High-Energy Synchrotron Source, NY. The data collection sofware for X-ray crystallography was HKL3000. Small-angle X-ray scattering (SAXS) data were collected at SYBILS beamline 12.3.1 at Lawrence Berkeley National Laboratory, CA. The data collection software was Blu-Ice/DSC for SAXS. The NMR spectroscopy data were obtained using a Bruker AVANCE 700 MHz spectrometer with the software TopSpin (version 3.6.0).
Data analysis	X-ray diffraction data were processed using HKL2000, COOT (version 0.9.5) and PHENIX (version 1.13). The small-angle X-ray scattering (SAXS) data were analyzed using ATSAS (version 2.4.2). The NMR spectroscopy data were analyzed using NMRPipe (version linux212_64) and NMRViewJ (version 9.2.b20). The analytical ultracentrifugation data were analyzed using the software SEDFIT (version 16.1c). The isothermal titration calorimetry data were analyzed using the Origin 7.0 software. All molecular representations were prepared using ChimeraX (version 1.5). Cell images were analyzed using ImageJ (version 1.53a) and CellProfiler (version 3.0). The statistical analyses for the cell biology were performed using GraphPad Prism (version 9.0.0).

For manuscripts utilizing custom algorithms or software that are central to the research but not yet described in published literature, software must be made available to editors and reviewers. We strongly encourage code deposition in a community repository (e.g. GitHub). See the Nature Portfolio guidelines for submitting code & software for further information.

Data

Policy information about availability of data

All manuscripts must include a data availability statement. This statement should provide the following information, where applicable:

- Accession codes, unique identifiers, or web links for publicly available datasets
- A description of any restrictions on data availability
- For clinical datasets or third party data, please ensure that the statement adheres to our policy

The crystallographic models and data have been deposited in the Protein Data Bank (PDB). The accession codes are 6MXX [https://doi.org/10.2210/pdb6mxx/pdb] (53BP1TT-UNC2991), 6MXY [https://doi.org/10.2210/pdb6mxy/pdb] (53BP1TT-UNC3351), 6MXZ [https://doi.org/10.2210/pdb6mxz/pdb] (53BP1TT-UNC3474), 6MY0 [https://doi.org/10.2210/pdb6my0/pdb] (53BP1TT-PN), and 8U4U [https://doi.org/10.2210/pdb8u4u/pdb] (53BP1TT-CC).

Human research participants

Policy information about studies involving human research participants and Sex and Gender in Research.

Reporting on sex and gender	Not applicable
Population characteristics	Not applicable
Recruitment	Not applicable
Ethics oversight	Not applicable

Note that full information on the approval of the study protocol must also be provided in the manuscript.

Field-specific reporting

Please select the one below that is the best fit for your research. If you are not sure, read the appropriate sections before making your selection.

☒ Life sciences ☐ Behavioural & social sciences ☐ Ecological, evolutionary & environmental sciences

For a reference copy of the document with all sections, see nature.com/documents/nr-reporting-summary-flat.pdf

Life sciences study design

All studies must disclose on these points even when the disclosure is negative.

Sample size	Sample sizes were not pre-determined statistically. In the case of immunofluorescence microscopy, a minimum of 100 cells were examined for each condition, a number chosen in accordance with established field standards and previous successful statistical analyses.
Data exclusions	No data were excluded from the analyses.
Replication	The biology experiments were repeated three times independently. All attempts at replication were successful.
Randomization	Randomization was not relevant to our study.
Blinding	For the cell biology, the investigators were blinded during data collection and analysis. One investigator recorded images of the cells while another investigator performed the quantifications.

Reporting for specific materials, systems and methods

We require information from authors about some types of materials, experimental systems and methods used in many studies. Here, indicate whether each material, system or method listed is relevant to your study. If you are not sure if a list item applies to your research, read the appropriate section before selecting a response.

Materials & experimental systems

n/a	Involved in the study
☐	☒ Antibodies
☐	☒ Eukaryotic cell lines
☒	☐ Palaeontology and archaeology
☒	☐ Animals and other organisms
☒	☐ Clinical data
☒	☐ Dual use research of concern

Methods

n/a	Involved in the study
☒	☐ ChIP-seq
☒	☐ Flow cytometry
☒	☐ MRI-based neuroimaging

Antibodies

Antibodies used

Flag M2 mouse monoclonal (Sigma-Aldrich, #F1804, lot SLCM4061) (dilution 1:2000); Beta-actin (C4) mouse monoclonal (Santa Cruz, #sc-47778, lot JO421) (dilution 1:5000); anti-HA-Tag (C29F4) monoclonal rabbit antibody (Cell Signaling Technology, #3724, lot 10) (dilution 1:1000); and anti-gammaH2A.X (Ser139) monoclonal mouse antibody (JBW301) (MilliporeSigma, #05-636, lot 3746841) (dilution 1:1000).

Validation

All antibodies used in this study were commercially available, validated by their respective manufacturers, and have been widely employed in the scientific literature. Furthermore, to ensure the specificity of the anti-Flag western blot (Figure 5a), a negative control was included, confirming that the observed signal on the western blot aligns with our expectations. Additionally, the bands were verified for their correct size.

Eukaryotic cell lines

Policy information about cell lines and Sex and Gender in Research

Cell line source(s)

The parental U2OS cells were from the American Type Culture Collection (ATCC).

Authentication

The modified cell lines were confirmed by immunoblotting.

Mycoplasma contamination

All cell lines were routinely confirmed mycoplasma negative by DAPI staining and PCR.

Commonly misidentified lines
(See ICLAC register)

None.
